# Modeling defects and plasticity in MgSiO_3_ post-perovskite: Part 3—Screw and edge [001] dislocations

**DOI:** 10.1007/s00269-017-0879-0

**Published:** 2017-03-09

**Authors:** Alexandra M. Goryaeva, Philippe Carrez, Patrick Cordier

**Affiliations:** UMET-Unité Matériaux et Transformations, CNRS, INRA, ENSCL, UMR 8207, Univ. Lille, 59000 Lille, France

**Keywords:** MgSiO_3_ post-perovskite, *D*″ layer, Dissociated dislocations, Mixed dislocation cores

## Abstract

In this study, we investigate the complex structure of [001] screw and edge dislocation cores in MgSiO_3_ post-perovskite at the atomic scale. Both [001] screw and edge dislocations exhibit spontaneous dissociation in (010) into two symmetric partials characterized by the presence of <100> component. In case of edge dislocations, dissociation occurs into ½<101> partials, while for the screw dislocations the <100> component reaches only 15%. Under applied stress, both [001](010) screw and edge dislocations behave similarly. Above the Peierls stress, the two partials glide together while keeping their stacking-fault widths (~11 and ~42 Å for the screw and edge dislocations, respectively) constant. The Peierls stress opposed to the glide of [001](010) screw dislocations is 3 GPa, while that of edge dislocations is 33% lower. Relying on the observed characteristics of the dislocation cores, we estimate the efficiency of [001](010) dislocation glide under the *P*–*T* conditions relevant to the lowermost mantle and demonstrate that dislocation creep for this slip system would occur in the so-called athermal regime where lattice friction for the considered slip system vanishes when the temperature rises above the critical *T*_*a*_ value of ~2,000 K.

## Introduction

One of the most prominent features of the *D*″ layer is the observation of well-developed seismic anisotropy (Vinnik et al. 1989; Lay et al. 1998; Panning and Romanowicz 2004). Several mechanisms can account for its development: crystal-preferred orientation (CPO) resulting from dislocation creep during flow, shape-preferred orientations (SPO) or alignment of melt inclusions. The possible contribution of CPO to seismic anisotropy has gained renewed attention since the discovery of the transition from MgSiO_3_ perovskite (bridgmanite) to the post-perovskite phase (Murakami et al. 2004; Oganov and Ono 2004; Tsuchiya et al. 2004) which exhibits a very anisotropic structure (space group *Cmcm, a* = 2.456 Å, *b* = 8.042 Å and *c* = 6.093 Å). Linking crystal plasticity to the development of CPO and seismic anisotropy requires information on slip systems activities (Wenk et al. 2011). So far this information is lacking. Indirect evidence of easy slip systems derived from the CPO observed during ultrahigh pressure experiments on MgSiO_3_ provides conflicting results (Merkel et al. 2007; Miyagi et al. 2010). Even in the low-pressure CaIrO_3_ analog material, which allows direct microstructural characterization, plastic anisotropy is insufficiently understood. Based on the TEM observations of dislocations in CaIrO_3_, the importance of [100](010) system seems well established; however, experimental evidence of deformation mechanisms along [010] and [001] directions in CaIrO_3_ is still scarce (Miyajima et al. 2006; Miyajima and Walte 2009). Also, one should mind that in terms of bonding and octahedral distortions, characteristics of CaIrO_3_ are not the same as in MgSiO_3_ and its closest MgGeO_3_ analog (Tsuchiya and Tsuchiya 2007; Hustoft et al. 2008; Kubo et al. 2008). First-principles studies also indicate that anisotropic character of elasticity (Tsuchiya and Tsuchiya 2007) and plasticity (Metsue et al. 2009) in CaIrO_3_ are different from those in silicate post-perovskite. All these ambiguities, associated with the analog approach in plasticity, call for direct investigation of MgSiO_3_ at relevant *P-T* conditions. Recent atomic-scale studies of [100] dislocations in MgSiO_3_ post-perovskite (Goryaeva et al. 2015b, 2016) emphasize the importance of [100] slip in (010) Mg-layers in this material. Alone, this single slip system cannot account for the plasticity of a crystalline aggregate and further mechanisms should be investigated. In this study, we focus on the dislocation activity that can contribute to slip along [001] direction in the silicate post-perovskite phase at 120 GPa.

Previous studies of generalized stacking-fault (GSF) energies, also called *γ*-surfaces (Carrez et al. 2007a; Metsue et al. 2009; Goryaeva et al. 2015a), have shown the presence of a stable stacking fault at half way when the crystal structure is sheared along [001] in the (010) plane. Thus, it is not surprising that early studies based on the Peierls–Nabarro (PN) model have suggested possible dissociation in (010) of [001] dislocations into two partials dislocations (Carrez et al. 2007a; Metsue et al. 2009). In addition to that, the recent *γ*-surface calculations (Goryaeva et al. 2015a) indicate that [001] dissociations can possibly exhibit a mixed character with the presence of <100> component, which could not be earlier described with the PN model (Carrez et al. 2007a, b). In this work, we investigate in detail the complex atomic structure of dissociated [001] screw and edge dislocations in MgSiO_3_ post-perovskite and the opposed lattice friction described through the Peierls stress. Furthermore, in order to constrain processes taking place in the lowermost mantle, we also estimate the efficiency of [001] dislocation glide under thermal activation.

## Models and methods

### Computational setup

**Fig. 1:**
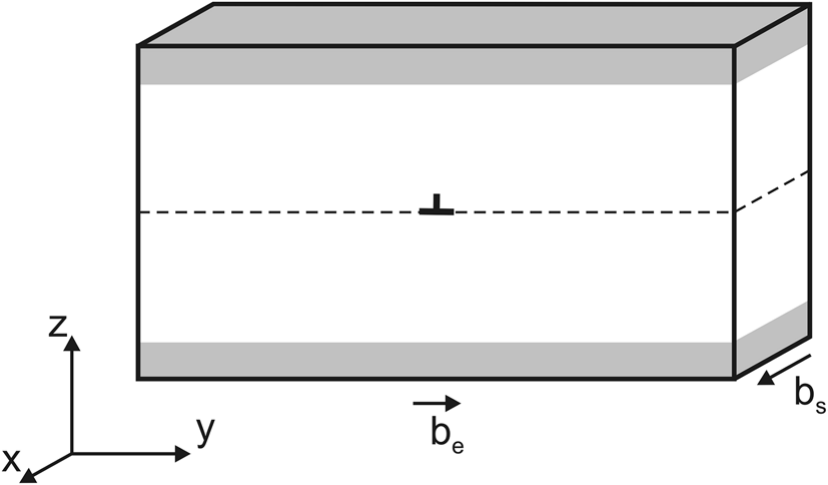
Geometry of atomic systems employed for dislocation modeling. Atoms at the *top* and *bottom* layers of the simulation cell, shown as *shaded areas*, are kept fixed; *dashed line* in the middle of the cell indicates location of the glide plane; vectors **b**_**e**_ and **b**_**s**_ correspond to Burgers vectors of edge and screw dislocations, respectively

The atomic systems used throughout this study to explore the dislocation core properties and dislocation motion are designed according to the methodology described by Hirel et al. (2014) for MgSiO_3_ perovskite (bridgmanite) which was also shown to be appropriate for modeling both edge and screw [100] dislocations in post-perovskite (see discussion in Goryaeva et al. 2015b). In this approach, a single dislocation is embedded into a simulation cell (Fig. 1) which is built to be fully periodic along the directions of the dislocation line (*x*) and of the dislocation glide direction (*y*). Along the *z* direction, normal to the glide plane, atoms at the bottom and at the top of the supercell (shown as shaded area in Fig. 1) are fixed to their regular positions and atomic relaxation is only allowed for the rest of the crystal. This enables mimicking an infinite perfect crystal and allows avoiding ineligible interactions between periodic replicas. The total width (along *z*) of the layer with fixed atoms is equal to the short-range cut-off distance of the potential, i.e., to 12 Å in our case. The simulation cell is as thin as a single unit cell parameter along *x* (parallel or normal to the Burgers vector **b** in case of screw and edge dislocations, respectively) which ensures the dislocation line to be straight and infinite while applying periodic boundary conditions. For screw dislocations, the supercell parameter aligned with *y* is tilted by ½**b** along *x* in order to keep periodicity along the dislocation glide direction. A special attention is given to the size of the system in order to minimize interactions of the dislocation with its periodic images. Thus, the size of the supercell is gradually increased until an equilibrium core configuration is achieved and also a convergence of the Peierls stress is reached. The typical size of a simulation supercell is ~350 Å along *y*, ~160 Å along *z*, and a single unit cell parameter along *x*. Such atomic arrays contain the order of 40,000–60,000 atoms depending on the unit length along the dislocation line direction *x*.

Employing such a large simulation cell is only possible while using semi-empirical pairwise potentials. In this study, atomistic modeling of dislocations is performed using the Buckingham form of a pairwise potential with the parameterization derived by Oganov et al. (2000) for MgSiO_3_ bridgmanite. Transferability of this parameterization was previously validated for MgSiO_3_ post-perovskite, including both ground state properties and modeling defects (Goryaeva et al. 2015a). Molecular statics calculations are carried out at *P* = 120 GPa and constant volume using the program package LAMMPS (Plimpton 1995), which relies on Ewald summation methods for Coulombic interactions. Energy minimization is performed using a conjugate-gradient algorithm followed by a Hessian-free truncated Newton algorithm until the maximum force on an atom drops below 10^−9^ eV/Å (1.602 × 10^−18^ N).

### Designing and analyzing dissociated screw and edge dislocation cores

Initial configurations of screw dislocations are created by imposing in a perfect crystal an isotropic displacement field corresponding to a single screw dislocation line with **b** = [001] (Hirth and Lothe 1982) using the command-line program Atomsk (Hirel 2015). Then, relaxation of the atomic positions (as described above) yields a dislocation core configuration counting anisotropic effects.

Edge dislocations are designed by building and joining along *z* (Fig. 1) two supercells, one of them containing an atomic extra half-plane. Structural relaxation of the joint atomic array gives rise to the edge dislocation (Osetsky and Bacon 2003; Bulatov and Cai 2006) with its Burgers vector aligned with *y* and its line lying along *x* (Fig. 1). In this study, edge dislocations are designed in such a way, that (010) glide plane is characterized with the lowest *γ*-energy (Goryaeva et al. 2015a), i.e., Si atoms in the dislocation core retain their favorable octahedral coordination. All atomic systems are constructed in such a way that charge neutrality and stoichiometry are preserved.

From the relaxed configuration of a dislocation core, one can compute the relative displacements of atoms near the core, also called disregistry *S*. For a dislocation core dissociated into two partials such as their dislocation lines are aligned with *x* and glide along *y* (Fig. 1), the corresponding *S*(*y*) can be described with the following function (e.g., Joos et al. 1994):

1 $$S(y)=\frac{b}{2}+\frac{b}{\pi }\left[ \alpha \times \arctan \left( \frac{y-{{y}_{1}}}{{{\zeta }_{1}}} \right)+(1-\alpha )\times \arctan \left( \frac{y-{{y}_{2}}}{{{\zeta }_{2}}} \right) \right],$$

where *b* is the full Burgers vector length; *α* ≤ 1 is a variable constant equal to 0.5 in case of symmetric dissociation; *y*_*i*_ is a location of the partial dislocation line (*i*) along the glide direction *y; ζ*_i_ is a half-width of the partial (*i*). The derivative d*S*(*y*)/d*y* describes the Burgers vector density *ρ*(*y*) in a given plane, and its full width at half maximum defines the width of the dislocation core, i.e., 2*ζ*. Distance between the maximum peaks of the *ρ*(*y*) depicts the dissociation distance *R* between the partial dislocation lines.

### Computing lattice friction

Lattice friction of a material is commonly quantified through the Peierls stress, i.e., the critical stress required to move a straight dislocation line from one Peierls valley to the next one in the absence of thermal activation (under the sole action of stress). In this work, dislocation glide is triggered by applying a simple shear strain to the simulation cell. The resulting force acting on the dislocation line is given by the Peach–Koehler equation (Peach and Koehler 1950):

2 $${\bf{F}_{\text{l}}}=\text{ (}\sigma \times {\bf{b}}\text{)}\times {\bf{l}},$$

where **F**_**l**_ is a force acting on a unit length of a dislocation line **l**; *σ* is the applied stress tensor resulting from straining the cell and **b** is the Burgers vector. All atomic systems are oriented in such a way that the dislocation line **l** is always aligned with *x* and glides along *y* (as indicated in Fig. 1). In order to initiate the dislocation glide, the *ε*_*xz*_ and the *ε*_*yz*_ strain components are gradually increased for screw and edge dislocations, respectively. To ensure quasi-static loading, after shear strain increment (of the order 10^−5^–10^−4^), the deformed atomic configuration is optimized using the setup for molecular static calculations described above. The Peierls stress is then defined as the critical stress needed to initiate dislocation glide.

## Results

### Stable [001] dislocation cores

#### Screw dislocations

**Fig. 2:**
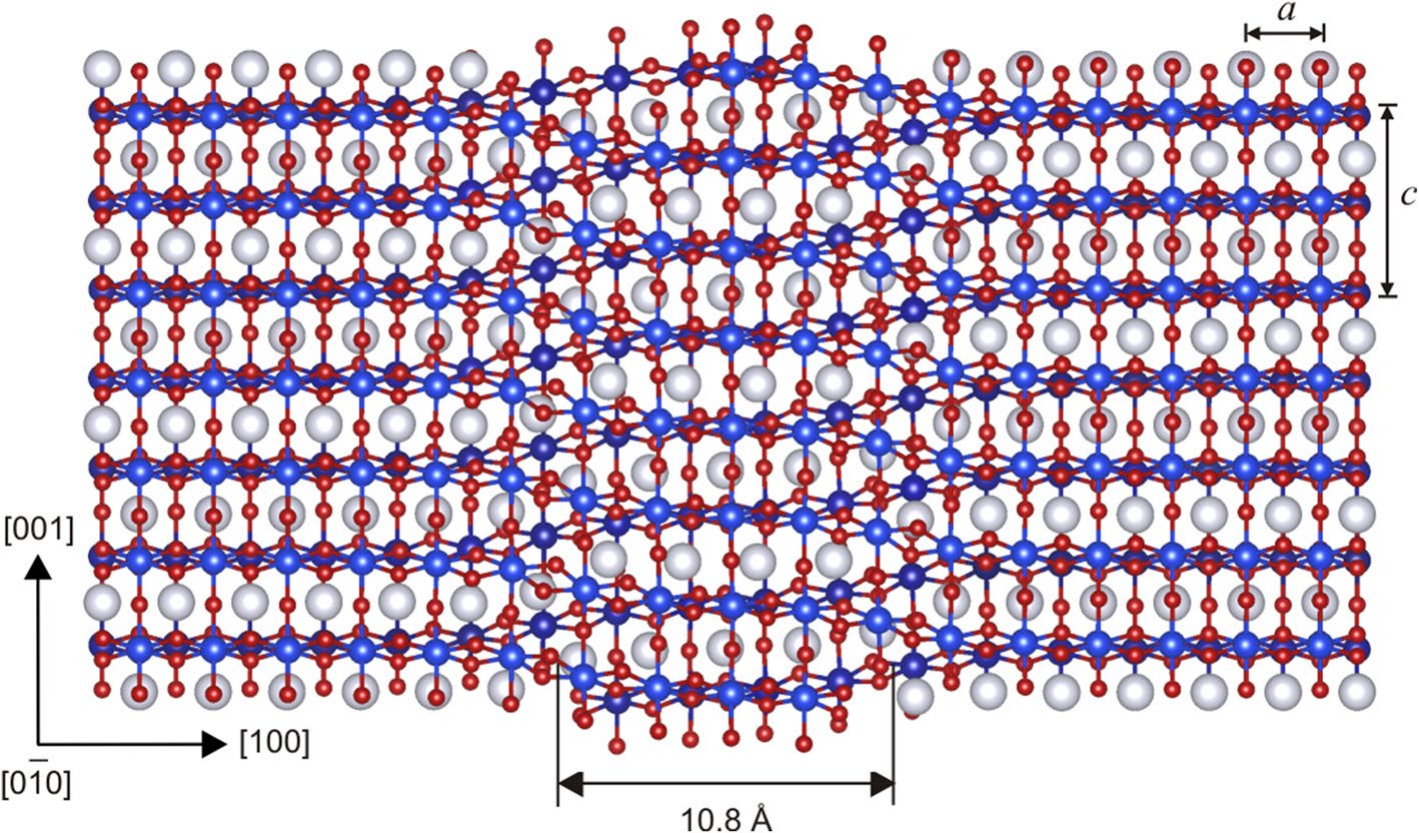
Atomic structure of the stable [001] screw dislocation core viewed in the (010) plane where it is contained (the dislocation *line* is running vertically). Si atoms are shown with *blue balls*, Mg—with *white*, and O—with *red*. The distance *R* between the two partials and the unit cell parameters *a* and *c* are indicated with *arrows*

**Fig. 3:**
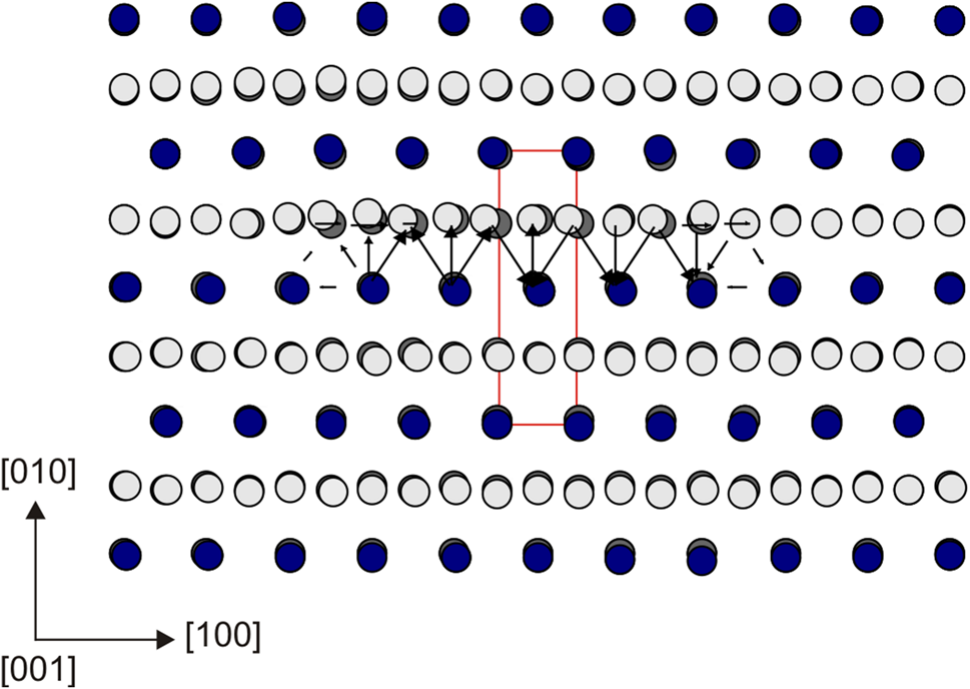
Differential-displacement (DD-plot) map of the [001] screw dislocation core viewed along the line direction (which coincides with the Burgers vector direction). The anion sublattice is left out; Si atoms are shown with *blue balls*; Mg atoms—with *light gray balls*; the unit cell—with *red rectangle*. Atomic positions in a perfect crystal are displayed in *dark gray* in order to highlight the presence of <100> edge component in the dislocation core. The *arrows* between neighboring atoms illustrate the [001] screw component (which is normal to the plane of representation) such as the lengths of *arrows* are proportional to the magnitude of the corresponding atomic displacements

**Fig. 4:**
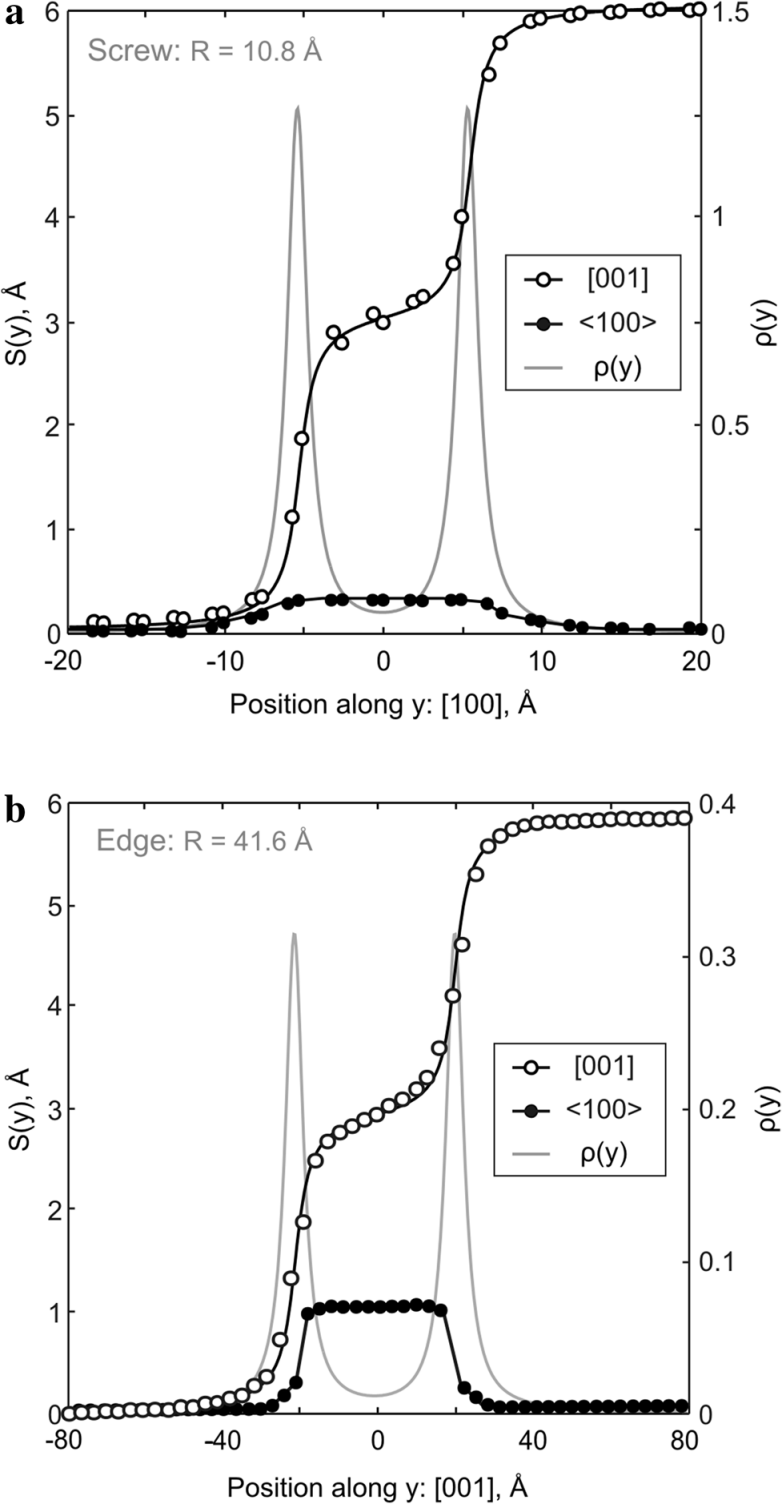
Disregistry function *S*(*y*) and the [001] Burgers vector density *ρ*(*y*) of stable screw (**a**) and edge (**b**) dislocations computed for the cation sublattice. *Open symbols* correspond to the [001] component; *black symbols*—to the <100> component; The Burgers vector density *ρ*(*y*) is plotted with *gray lines*

In agreement with predictions from previous GSF studies (Carrez et al. 2007a; Metsue et al. 2009; Goryaeva et al. 2015a), we observe spontaneous dissociation of perfect [001] Volterra dislocations into two partials separated by a stacking fault in (010). The atomic structure of the relaxed screw dislocation core is illustrated in Fig. 2 where the stacking fault between the two partials can be clearly distinguished. The corresponding differential-displacement (DD) map is presented in Fig. 3 where the crystal structure is oriented in such a way that the dislocation is now seen along its line. The relative displacements of neighboring atoms produced by the [001] screw dislocation along the line direction are represented by arrows scaling with respect to the displacement amplitude. The largest atomic displacements are localized in (010) between the Mg- and Si-layers. Quantitative characteristics of the dislocation core (Table 1) are derived from its disregistry function *S*(*y*) computed from Eq. (1) for the cation sublattice and its derivative *ρ*(*y*) describing distribution of the [001] Burgers vector density in the glide plane. Symmetric shapes of both functions (Fig. 4a) indicate that splitting occurs into two geometrically identical partials. The core spreading of each partial is very narrow and characterized by the half-width *ζ* close to 1 Å (Table 1). The distance *R* between the partials is estimated as the spacing between the two maximum peaks *ρ*(*y*) and found to be 10.8 Å (Figs. 2, 4a) which is close to 4 × *a* unit cell parameters. It should be noted that in addition to the [001] screw component, the dislocation core exhibits the presence of ~15% <100> edge component (Fig. 4a), i.e., presence of small displacements perpendicular to the dislocation line. On the DD-plot map (Fig. 3), such atomic displacements in the dislocation core can be clearly seen within the Mg sublattice by superposing the atomic layout in the dislocation core with that in a perfect crystal (represented in dark gray in Fig. 3). Alternatively, from Fig. 2, one can notice that, out of the dislocation core, Mg atoms are perfectly aligned along the [100] direction with vertical bonds between silicon and apical oxygen atoms (in the perfect crystal both Mg and apical O occupy *4c* Wyckoff positions), while within the stacking-fault area, the Mg sublattice is slightly displaced towards the empty spacing between Si–O bonds due to the presence of the small <100> edge component.

**Table 1:** Computed characteristics of the dissociated [001](010) dislocation cores

	Dislocation line orientation	
	[001]	[100]
Geometry and stacking-fault energy		
Length of partial Burgers vector **b**_**p**_ (Å)	3.05	3.27
Distance *R* between the two partials (Å)	10.8	41.6
Half-width *ζ* of a partial (Å)	1.05	3.07
Angle *θ* between **l**_**p**_ and **b**_**p**_ (°)	±7	±68
Stacking-fault energy *γ* (J/m^2^)	2.85	1.80
Lattice friction		
Peierls stress *σ*_p_ (GPa)	3	2
Periodicity of the Peierls potential *a*′ (Å)	2.5	3.0

Regardless of the dislocation line orientation, partial dislocations always have a mixed character. Depending on the line orientation, dissociation follows different paths and involves different partial Burgers vectors **b**_**p**_ (see Fig. 8 and the text for details)

#### Edge dislocations

**Fig. 5:**
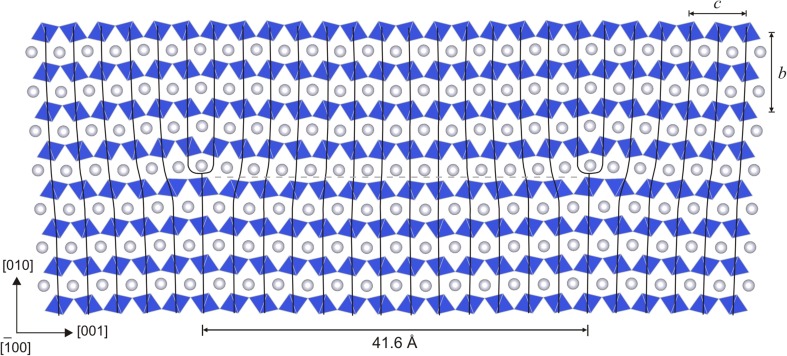
Atomic structure of the stable [001] edge dislocation core viewed along the [100] direction. SiO_6_ octahedra are shown in *blue*; Mg atoms—with *white balls*. The distance *R* between the two partials and the unit cell parameters *b* and *c* are indicated with *arrows*

Taking into account that [001] screw dislocations dissociate in the (010) plane and that their dissociation in any other plane has been never observed, we focus on [001](010) edge dislocations only. From the atomic structure of the relaxed [001](010) edge dislocation core (Fig. 5), one can notice that it also splits into two symmetric partials separated by a widespread stacking fault where orientation of Si-octahedra located below and above the glide plane resembles a mirror reflection of each other. Such a configuration can be produced by shearing the upper (or lower) part of the crystal by ½<101> in (010). In other words, this atomic arrangement explicitly reproduces the structural geometry of the metastable stacking fault in the middle of the (010) *γ-*surface cutting Mg–O bonds only (see Fig. 3c in Goryaeva et al. 2015a). The disregistry function *S*(*y*) of the observed dislocation core (Fig. 4b) confirms its mixed character and indicates simultaneous presence of both ½[001] edge and ½<100> screw components. The estimated distance *R* between the partials is 41.6 Å (Figs. 4b, 5) which is close to 7 × *c* unit cell parameters and almost four times larger than that of the stable [001] screw dislocation. The half-width *ζ* of each partial is found to be about 3 Å (Table 1), close to ½*b*.

### Evolution of dislocation core characteristics with dissociation distance

As both screw and edge components of [001] dislocations appear to dissociate in (010), we also seek for structural optimization of metastable dislocation cores with different separation distance between the partials. Hence, [001] screw dislocations are alternatively designed by imposing separately the two ½[001] pure screw or mixed ½<101> partials in such a way that the distance between them along [100] varies from 5 to 50 Å. In case of edge dislocations, our initial building scheme restricts the search for metastable cores to those encountered during the molecular static minimization steps.

Accounting for a change in dissociation width *R* in various metastable configurations with respect to that in the stable dislocation core, the associated energy increase ∆*W* should scale with the following expression (Hirth and Lothe 1982):

3 $$\frac{\Delta W}{L}~=~\gamma \left| {{R}_{\text{SF}}}-R_{\text{SF}}^{\text{eq}} \right|~~-~~\frac{\mu }{2\pi }({\bf {b}_{1}}\times {\bf {l}_{1}})({\bf {b}_{2}}\times {\bf {l}_{2}})\ln \frac{{{R}_{\text{SF}}}}{R_{\text{SF}}^{\text{eq}}}~~-~~\frac{\mu }{2\pi (1-\nu )}[({\bf {b}_{1}}\times {\bf {l}_{1}})({\bf {b}_{2}}\times {\bf {l}_{2}})]\ln \frac{{{R}_{\text{SF}}}}{R_{\text{SF}}^{\text{eq}}},$$

where ∆*W*/*L* is the increase in energy per dislocation unit length with respect to the equilibrium configuration characterized by a dissociation width $R_{\text{SF}}^{\text{eq}}$; *R*_SF_ is the extension of a perfect stacking fault between the two partials; *µ* is the anisotropic shear modulus; *ν* is the Poisson ratio; **b**_**i**_ and **l**_**i**_ are the partial Burgers vectors and the dislocation line vectors, respectively. It should be noted, that the separation distance *R*, defined in this work as the spacing between the two maximum peaks of the Burgers vector density *ρ*(*y*), actually describes the spacing between the two partial dislocation lines. Therefore, we define the width of a perfect stacking fault for each dislocation core as *R*_SF_ = *R* − 2*ζ* (where *ζ* is a half-width of each partial). According to Eq. (3), the energy increase ∆*W*/*L* results from misbalance between the elastic interaction energy of partial dislocations (the last two terms) and the stacking-fault energy (the first term).

**Fig. 6:**
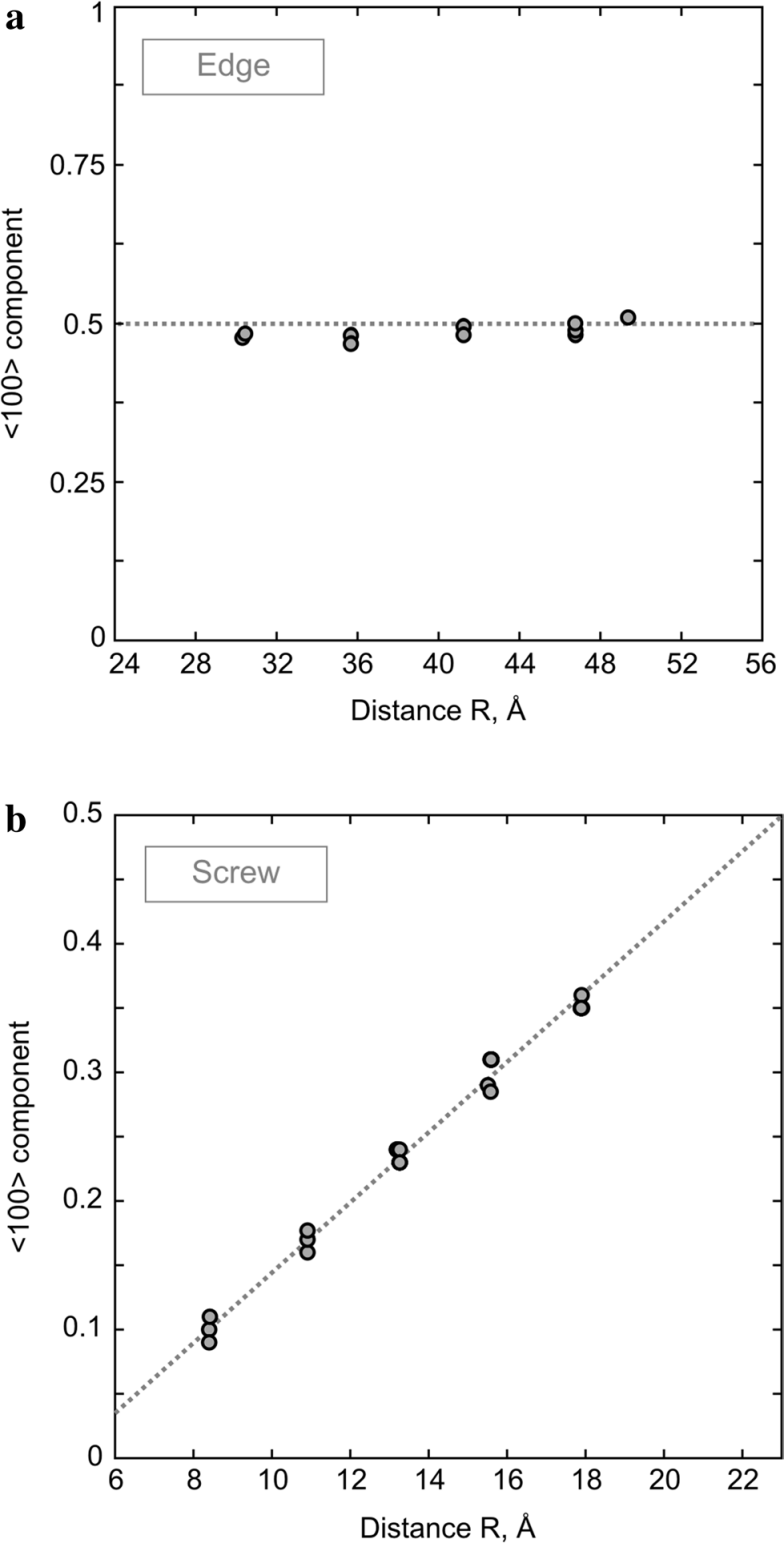
Evolution of the <100> component in [001] edge (**a**) and screw (**b**) dislocation cores with increasing distance *R* between the partial dislocation lines

In case of [001](010) edge dislocations, the metastable core configurations consistently exhibit a planar dissociation of [001] Burgers vector into two partial dislocations separated by a distance *R* varying from 30 to 50 Å. These cores are characterized by the presence of a ½<100> screw component which does not depend on *R* (Fig. 6a). This indicates that all these dislocation cores are associated with the same *γ-*energy corresponding to the ½<101>(010) stacking fault. As expected from elasticity, the observed configurations of [001] screw dislocations are less extended than those of edge dislocations. The estimated distance *R* between the screw partials varies from 8.5 to 18 Å. In contrast to the edge dislocations with the regular stacking-fault geometry (Fig. 6a), <100> component in [001], screw dislocation cores increase linearly with *R* from 10 to 35% of the [100] lattice repeat (Fig. 6b). This means that all detected screw dislocation cores are characterized by different *γ*-energies, i.e., they are associated with various stacking-fault configurations.

**Fig. 7:**
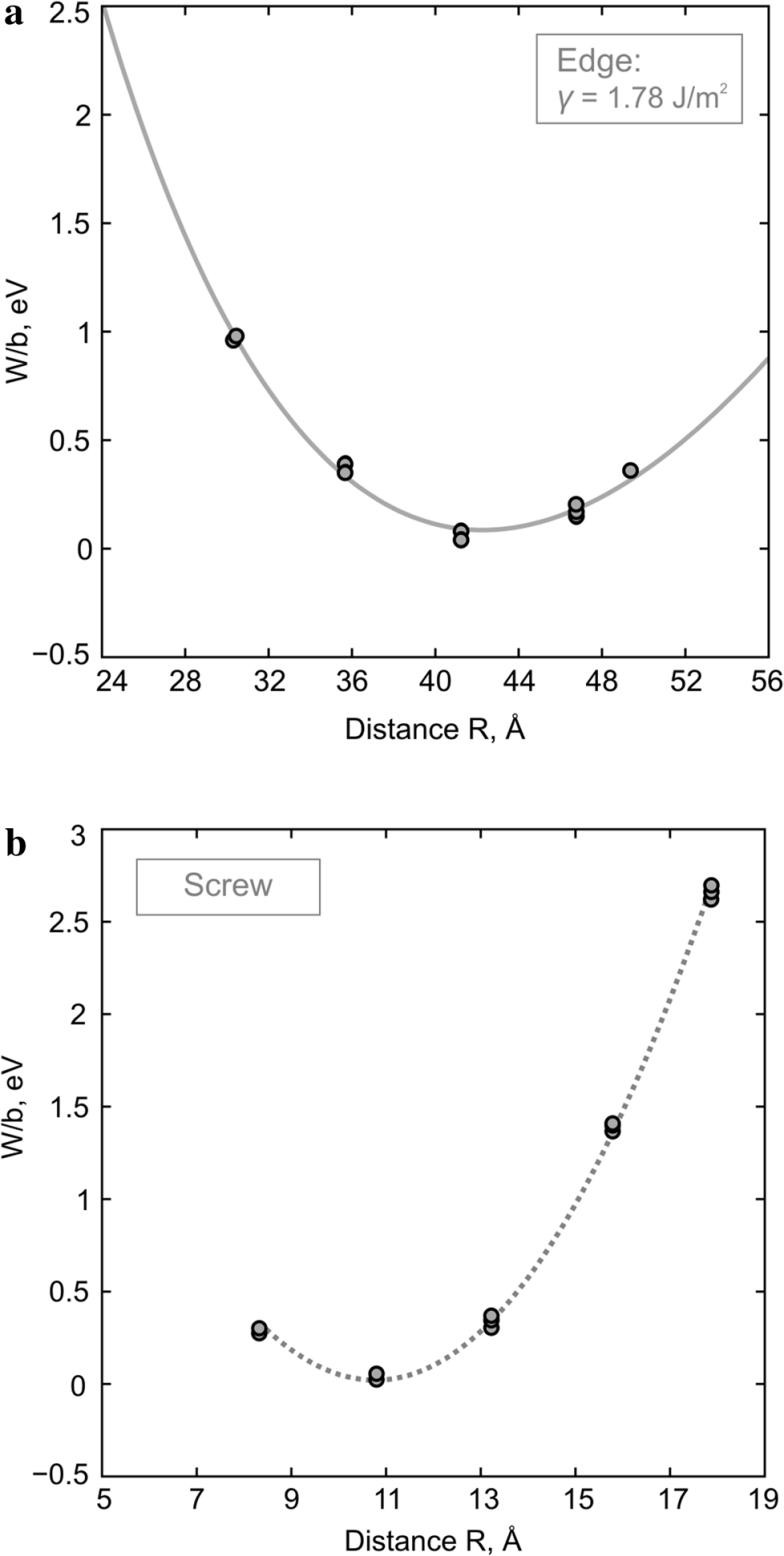
Estimated energies of the observed [001] edge (**a**) and screw (**b**) dislocation cores (once the minimum energy is extracted) shown as a function of the dissociation distance *R* between the partial dislocation lines. Energies of the edge dislocation cores (**a**) are fitted with the elastic equilibrium Eq. (3) illustrated with the *solid gray line*

Dealing with significantly extended dislocation cores prevents us from using fully periodic atomic systems for estimating their energies. Thus, we verify the atomic structure of the “frozen” area (shown with gray in Fig. 1) to be strictly the same for all configurations and compute potential energy of the atomic array not included into this region. Dimensions of the supercells were gradually increased until there is no size effect on computed energies. The estimated energy values (once the minimum energy is extracted) for edge and screw dislocations are presented in Fig. 7a, b as a function of the separation distance *R* between the partials. The minimum energy configurations correspond to the edge and screw dislocations with *R* = 41.6 and 10.8 Å, respectively, i.e., to the stable dislocation cores described in the previous sections.

Based on the observed geometric and energetic characteristics of dissociated edge dislocation cores associated with the ½<101>(010) stacking fault, one can estimate the corresponding *γ*-energy from the elastic equilibrium Eq. (3). Fitting the dislocation core energies computed at the atomic scale (Fig. 7a) with Eq. (3) employing the estimated *R*_SF_, *µ* = 184 GPa calculated from elastic constants and anisotropic Poisson ratio *ν* = 0.454 determined using GULP (Gale and Rohl 2003) provides *γ* = 1.78 J/m^2^. This value is slightly (~15%) lower than the corresponding energy predicted from classic *γ*-surface calculations performed with the same pairwise potential parameterization (Goryaeva et al. 2015a).

In case of screw dislocations, all the dislocation cores with different dissociation widths are characterized by different stacking-fault configurations between the partials, involving different *γ*-energies. Therefore, for each dislocation core configuration, the corresponding *γ*-energy is computed with anisotropic elasticity within the Stroh formalism (Stroh 1958), relying on the derived geometric characteristics of the dislocation cores as it is further described in the “Discussion” section.

### Dislocation glide

In order to estimate the Peierls stress *σ*_p_ associated with [001] dislocation glide, the relaxed configurations of stable screw (*R* = 10.8 Å) and edge (*R* = 41.6 Å) dislocation cores are investigated under applied shear stress. Glide of dissociated dislocations always occurs in the plane where dislocation core spreads, i.e., in (010) in our case.

Applying simple shear strain *ε*_*xz*_ to the screw dislocation core increases stress in (010) normal to *z* and triggers glide along [100] aligned with the *y* axis (Fig. 1). The dislocation remains immobile until the stress reaches a critical value corresponding to the Peierls stress *σ*_p_ = 3 GPa, when the dislocation core moves by one [100] lattice repeat. The two partials glide together and tend to keep constant both the separation distance *R* = ~11 Å and their half-widths *ζ* = ~1 Å (Table 1). In other words, the geometry of the dissociated [001](010) screw dislocation core does not exhibit any substantial changes during dislocation glide.

The onset of motion of a stable edge dislocation is studied by applying a simple shear strain *ε*_*yz*_ in order to enhance dislocation glide along [001] aligned with *y* in (010) plane normal to *z* (Fig. 1). As expected, edge dislocations, being much extended, are more mobile than screw dislocations and start gliding at *σ*_p_ = 2 GPa. Their behavior under applied stress is similar to that observed for the screw dislocations, i.e., both partials move together by successive steps of ½[001] lattice parameter while keeping constant distance *R* of ~42 Å and half-widths *ζ* of ~3 Å (Table 1).

## Discussion

### Dissociation process

**Fig. 8:**
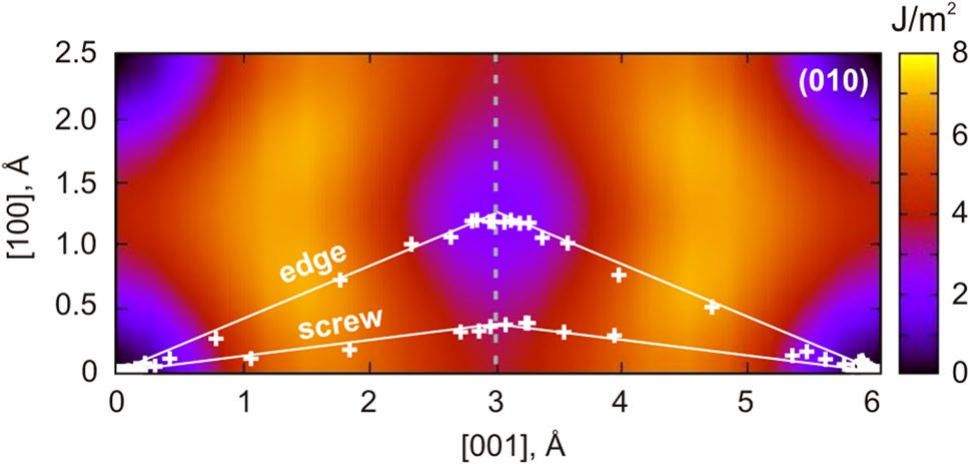
Dissociation paths of the stable [001] edge and screw dislocations in the (010) plane shown in projection to the corresponding *γ*-surface energy map (Goryaeva et al. 2015a). Location of *white crosses* on the map is extracted from the exact atomic positions in the stable [001] dislocation cores (Figs. 2, 4, 5); *solid white lines* are the visual guides depicting dissociation path for each dislocation type; *dashed gray line* in the middle of the map indicates ½[001](010) stacking fault with the <100> component varying from 0 to ±50%. The maximum value of ½<100> is reached for the edge dislocations, while for the screw dislocations <100> component is almost three times smaller

Atomistic modeling of both [001] screw and edge dislocations show that they dissociate in (010) into two symmetric partials separated by a stacking fault. Both dislocations exhibit mixed characters and are characterized by the presence of <100> components. However, in case of edge dislocations, dissociation occurs into two ½<101> partials, while for the screw dislocations, dissociation [001] → ½[101] + ½[−101] involves high-energy configurations (Fig. 7b) and, therefore, it has been never observed. The dissociation path of stable screw and edge dislocation cores is presented in Fig. 8 in projection to the corresponding *γ*-energy map from previous GSF calculations (Goryaeva et al. 2015a). Increasing <100> component along the ½[001](010) stacking fault (indicated with dashed gray line in Fig. 8) results in decrease in *γ*-energy and, consequently, to increase in the stacking-fault width between the partials. Such dependence between the stacking-fault energy and dissociation distance explains the observed relation between the <100> component and the distance *R* in various metastable configurations of dissociated [001](010) screw dislocation cores (Fig. 6b). At the same time, dissociation of the edge dislocations appears to be large enough to incorporate invariant ½<100> component (Fig. 6a).

According to the elastic theory, being characterized with the same stacking-fault energy *γ*, the size of screw and edge dislocations should scale with a ratio of 1/(1 − *ν*). Thus, [001] screw dislocations with ½<100> edge component would be characterized by *R* close to 23 Å which agrees well with the linear trend (Fig. 6b) describing evolution of <100> component in [001] screw dislocations with increasing distance *R* between the partials.

Dissociation of [001] screw and edge dislocations in (010) plane was previously investigated from the first-principles based on the Peierls–Nabarro (PN) model (Carrez et al. 2007a, b). However, these early studies were focused on pure screw and pure edge dislocation cores without taking into account the presence of <100> component, i.e., mixed [001] dislocation cores have never been modeled before. As it can be expected, the predicted dissociation width of 16.2 Å for pure [001](010) edge dislocations (Carrez et al. 2007a, b) is significantly lower, than 41.6 Å found in the present work for the mixed dislocation core. Nevertheless, for the screw dislocations characterized by the presence of only 15% <100>, both the separation distance *R* = 10.8 Å and the half-width of each partial *ζ* = 1.0 Å (Table 1) compare well with the values *R* = 9.9 Å and *ζ* = 1.1 Å predicted by the PN model for the pure [001] screw dislocation core (Carrez et al. 2007a, b).

### Stacking-fault energies

In case of dissociated dislocations, stacking-fault energies represent an essential parameter influencing their dissociation width. At the atomic scale, they are commonly estimated from GSF calculations as the excess energy associated with a stacking fault created by imposing a rigid-body shear (Vitek 1968). In this study, stacking-fault energies *γ* are alternatively computed through fitting the potential energies of several metastable ½<101>(010) edge dislocation cores with different dissociation width (Fig. 7a) according to isotropic elasticity (3). The energy fit provides *γ* = 1.78 J/m^2^ which is slightly lower than the corresponding value from previous *γ*-surface calculations based on the same potential model (Goryaeva et al. 2015a).

**Fig. 9:**
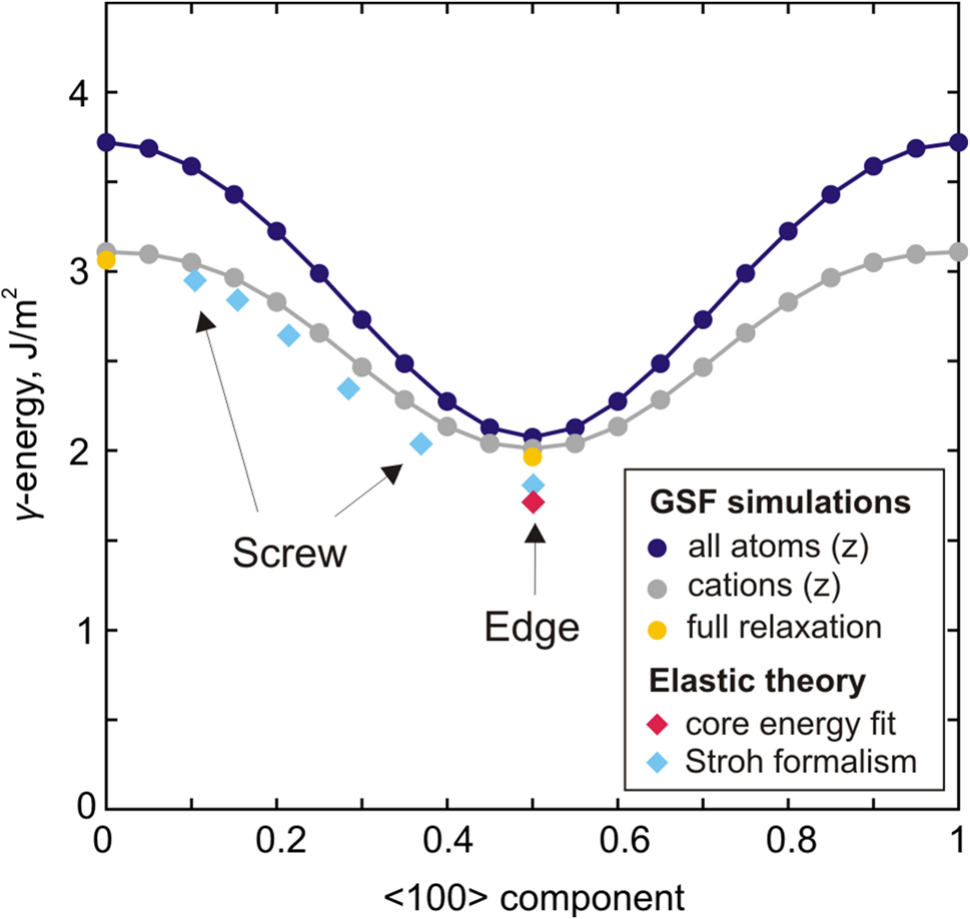
Evolution of the ½[001](010) stacking fault *γ*-energy along the [100] direction estimated with *γ*-surface (GSF) calculations and from elastic theory (relying on the dislocation core geometries determined in this study)

In conventional GSF calculations all atoms are allowed to move perpendicular to the glide plane only (in order to avoid the perfect crystal recovery) which inevitably leads to overestimation of energies. In order to reduce this effect, we additionally performed the same kind of calculations where degrees of freedom are restricted for cations only while oxygen atoms are allowed to relax fully in all directions. This leads to a decrease in *γ-*surface energy associated with ½[001](010) stacking fault by ~0.6 J/m^2^ (Fig. 9); however, while the <100> component increases (following the dashed gray line in Fig. 8), this effect subsists and almost vanishes for the ½<101>(010) configuration (Fig. 9). Allowing full relaxation for all atoms in highly symmetric configurations with ½[001] and ½<101> shift provides energies very close to those obtained while restricting only cations (Fig. 9). Therefore, the latest can be considered as the reference *γ-*energy to compare with.

Relying on structural information derived from the performed atomic-scale dislocation modeling, we also compute the related *γ-*energies with anisotropic elasticity using the DISDI code (Douin et al. 1986) based on the Stroh formalism (Stroh 1958). The estimated energies for five different configurations of [001] screw dislocations with <100> edge component varying from 10 to 35% and for the stable configuration of ½<101> edge dislocation are presented in Fig. 9. These energies are consistent with those from GSF calculations where degrees of freedom were restricted only for cations. The corresponding *γ*-energies for stable screw (15% of < 100 > component) and edge dislocations (50% of <100> component) are 2.85 and 1.80 J/m^2^, respectively, as reported in Table 1.

### Lattice friction

Lattice friction of a material can be described through the Peierls stress or through the energy barrier that a straight dislocation line should overcome in order to move from one equilibrium position to the next one in absence of thermal activation. In this work, we compute the lattice friction as the Peierls stress opposed to the glide of [001] dislocations in (010) plane. Under applied shear stress, both [001](010) screw and edge dislocations exhibit a similar behavior. The two partials glide together while keeping the stacking-fault width constant. Being characterized by a larger Peierls stress of 3 GPa, the mobility of screw dislocations is expected to govern plastic strain for this slip system. However, it is worth noticing that, in contrast to [100] dislocations in post-perovskite (Goryaeva et al. 2015b), where the Peierls stress *σ*_p_ is found to be one order of magnitude lower for edge dislocations than for screw, *σ*_p_ of edge dislocations is only 33% lower in case of the [001](010) system (Table 1).

Relying on the observed characteristics of dislocation cores and their response to applied shear (evolution of the core configuration, Peierls stress), one can estimate the corresponding Peierls barrier. Dislocation motion by successive steps of [100] and ½[001] lattice repeats implies periodicity of the Peierls potential, denoted hereafter as *a*′, to be equal to *a* = 2.5 Å and *c*/2 = 3 Å unit cell parameters (Table 1) for screw and edge dislocations, respectively. Simultaneous motion of both partials allows assuming a simple sinusoidal shape of the Peierls potential. Therefore, the maximum energy barrier for each partial can be defined as (Koizumi et al. 1993): $V_{\text{p}}^{\text{max}}=\frac{1}{\pi }{a}'{{b}_{\text{p}}}{{\sigma }_{\text{p}}}$, where *a*′ is periodicity of the Peierls potential; *b*_p_ is the partial Burgers vector length; and *σ*_p_ is the Peierls stress. The estimated $V_{\text{p}}^{\max }$ values are 46 meV/Å for screw dislocations and 39 meV/Å for edge dislocations. Based on a similar approach, one can find that the corresponding $V_{\text{p}}^{\max }$ energies for the [100](010) system in MgSiO_3_ post-perovskite (Goryaeva et al. 2015b, 2016) are 15 and 2 meV/Å for screw and edge dislocations, respectively. Although higher than for the [100] dislocations, lattice friction opposed to the [001] dislocation glide in (010) is still very low. Together, the high mobility of [100] and [001] dislocations within the MgO layers in the post-perovskite clearly emphasize the importance of (010) for plastic deformation of this phase. For these slip systems, lattice friction is comparable with that in MgO periclase (0.9 and 2 GPa for ½[110](100) and ½<110>{110} systems, respectively) computed from the first principles at the confining pressure of 100 GPa (Cordier et al. 2012). Compared to the lattice friction computed for the easiest slip systems in MgSiO_3_ bridgmanite (Kraych et al. 2016a) using the same pairwise potential parameterization, the Peierls barriers opposed to the dislocation glide in (010) in the post-perovskite are one order of magnitude lower. Such theoretical evidence of the remarkably low lattice friction in silicate post-perovskite in conjunction with the fast diffusion in this phase (Ammann et al. 2010) suggest a pronounced rheological contrast between the weak *D*″ and the overlying mantle with high-lattice friction bridgmanite as a dominant constituent.

### Implications: [001](010) dislocation glide under the lower mantle conditions

Atomistic modeling of [001](010) dislocations gives access to dislocation core geometries, associated *γ*-energies and to the opposed lattice friction, denoted above as the Peierls stress *σ*_p_ at which a dislocation starts gliding at 0 K. At finite temperature, the actual motion of a dislocation from one Peierls valley to another is assisted both by stress and thermal activation. The corresponding mechanism involves nucleation and propagation of kink-pairs. The dislocation does not move as a straight line, but partly bows out over the Peierls potential. For a dissociated dislocation, the enthalpy ∆*H* of this process consists of four terms and can be described as follows (Mitchell et al. 2003; Ritterbex et al. 2015):

4 $$\Delta H=\Delta {{E}_{\text{el}}}+\Delta P+\Delta {{W}_{\text{sf}}}-{{W}_{\sigma }},$$

where ∆*E*_el_ is the increase in elastic energy caused by the increase in length of the dislocation line; ∆*P* is the change in Peierls energy of the line portion which leaves the Peierls valley; ∆*W*_sf_ is the energy cost associated with the change in the stacking-fault energy *γ*; and, finally, *W*_σ_ is the negative contribution due to the work of the applied stress.

The kink-pair formation mechanism is commonly investigated based on the line tension (LT) model (Guyot and Dorn 1967), the kink–kink (KK) interaction model (Seeger and Schiller 1962) or on the elastic interaction model (Koizumi et al. 1993, 1994). These models only differ by the way the ∆*E*_el_ term is calculated. Employing any of them in conjunction with direct atomic-scale modeling of kink-pairs on dissociated dislocation cores requires calculations which are beyond the scope of the present study. However, one can still estimate the range of the so-called athermal temperature *T*_*a*_ based on the computed characteristics of straight [001] dislocations (core geometry, *γ*-energy and Peierls barrier) allowing to do some reasonable assumptions. The athermal temperature *T*_*a*_ corresponds to a critical temperature above which thermal activation is sufficient to overcome lattice friction and therefore incipient plastic deformation becomes temperature independent (e.g., Cordier et al. 2012). Below this critical temperature, plastic deformation is mostly controlled by lattice friction, while above *T*_*a*_, i.e., in the athermal deformation regime, plastic flow is governed by the microstructure produced by dislocation–dislocation interactions and occurs at rather constant and low stress (Kubin 2013).

As it has been shown by Koizumi et al. (1993), when the ratio *σ*_p_/*μ* is close to 10^−2^, the ∆*P* term contributes only about 1% to the total kink-pair formation enthalpy ∆*H*. For the [001] screw dislocations, this ratio is equal 1.6 × 10^−2^, which indicates that the positive contribution of the Peierls potential can be neglected.

The energy term ∆*W*_sf_ is relevant only in case of the uncorrelated nucleation (Ritterbex et al. 2015) when kink-pairs nucleate independently on leading and trailing partials which locally changes the stacking-fault width and, consequently, its energy. Involving additional energy cost, this process is likely to occur only above a critical stress *σ* ^crit^, while below this value only correlated nucleation (both kink-pairs nucleate simultaneously such as their centers coincide) takes place without changing the stacking-fault width along the partials. The critical stress can be estimated as (Möller 1978): ${{\sigma }^{\text{crit}}}=\frac{\pi {{\gamma }^{2}}{a}'}{\beta \mu b_{\text{p}}^{3}}$, where *γ* is the stacking-fault energy; *a*′ corresponds to the periodicity of the Peierls potential; *b*_p_ is the partial Burgers vector length; and *β* is a function of the angles *θ*_*i*_ between the partial Burgers vectors **b**_**i**_ and the dislocation lines **l**_**i**_ defined as *β* = cos*θ*_1_cos*θ*_2_ + sin*θ*_1_sin*θ*_2_/(1 − *ν*), where *ν* is the Poisson ratio. Employing previously defined *ν* = 0.454, *γ* = 2.85 J/m^2^ and *θ*_*i*_ = ±7° for the [001] screw dislocations (Table 1), the critical stress *σ* ^crit^ is found to be ~1.3 GPa which is well above the stresses expected in the lower mantle. Therefore, in case of the [001](010) screw dislocations only correlated kink-pair nucleation is expected to occur in mantle conditions and, therefore, the ∆*W*_sf_ term can be disregarded.

Based on all these assumptions, with no stress applied, the formation enthalpy *H*_2*k*_ (*σ* = 0) of a rectangular kink-pair with large width *w* (such as *w* > > *h*, where *h* = *a*′ is the kink height) on each partial can be estimated as (Möller 1978; Hirth and Lothe 1982):

5 $${{H}_{2k}}=\frac{\mu {a}'}{2\pi }\left[ b_{s}^{2}\left( \frac{1}{1-\nu }\ln \frac{{{a}'}}{e\rho }-1 \right)+b_{e}^{2}\left( \ln \frac{{{a}'}}{e\rho }-\frac{1}{1-\nu } \right) \right]$$

where *μ* is the anisotropic shear modulus; *a*′ is the periodicity of the Peierls potential; *b*_s_ and *b*_e_ are the screw and edge components of *b*_p_, respectively; *ν* is the Poisson ratio; *e* is the Euler’s number; and *ρ* is the cut-off distance commonly taken as 5–10% of the Burgers vector. The *H*_2*k*_ value defined using Eq. (5) is very sensitive to the choice of *ρ* (Koizumi et al. 1993). Thus, varying the cut-off *ρ* from 5 to 10% of the partial Burgers vector *b*_p_ results in deviation of the *H*_2*k*_ values from 9.7 to 4.2 eV. It should be noted that independently of *ρ*, contribution of the edge component into *H*_2*k*_ does not exceed 0.2% due to the very small *b*_e_ = *b*_p_sin*θ*; hence, the second term in Eq. (5) can be neglected. Based on the model of Koizumi et al. (1994) and results of the recent atomic-scale calculations in conjunction with the elastic interaction model (Kraych et al. 2016a), the kink-pair enthalpy *H*_2*k*_ at zero stress can be approximated as $\mu b_{\text{p}}^{3}\sqrt{{{\sigma }_{\text{p}}}/\mu }$, which provides *H*_2*k*_ = 4.15 eV consistent with *ρ* = 0.1 *bp* in Eq. (5). Relying on this value, the estimated *V*_p_^max^ = 46 meV/Å is indeed close to 1% of *H*_2*k*_ in agreement with Koizumi et al. (1993). As it was shown above, motion of a dissociated [001] screw dislocations is expected to occur through correlated kink-pair nucleation; therefore, the energetic cost associated with the kink-pair formation on both partials can be set as *H*_4*k*_ = 2*H*_2*k*_ = 8.3 eV.

Combining the Orowan’s equation with the assumption of dislocation velocities controlled by kink-pair nucleation, the shear strain rate $\dot{\varepsilon }$ can be expressed as a function of stress *σ* (Guyot and Dorn 1967):

6 $$\dot{\varepsilon }={{\rho }_{{m}}}b\bar{v}=\frac{{{\rho }_{{m}}}L{a}'{{b}^{2}}{{\nu }_{D}}}{2{{w}^{2}}}\exp \left( \frac{-2{{H}_{2k}}(\sigma )}{kT} \right),$$

where *ρ*_*m*_ is the density of mobile dislocations; $\bar{v}$ is the dislocation glide velocity; *b* is the full Burgers vector length; *a*′ stands for periodicity of the Peierls potential; *w* corresponds the kink-pair width; $L=1/\sqrt{{{\rho }_{{m}}}}$ describes the average length of dislocation lines ; *ν*_*D*_ is the Debye frequency; $\sigma$ is the resolved shear stress and *k* is the Boltzmann constant. In conjunction with Eq. (6), we assume that the kink-pair enthalpy 2*H*_2*k*_ scales linearly with *kT* (Kubin 2013). Then, once 2*H*_2*k*_ (*σ* = 0) is defined, the athermal temperature can be estimated, while solving Eq. (6) for the case of zero applied stress, which provides ${{T}_{{a}}}=\frac{2{{H}_{2k}}}{kC}$, where $C=\ln \frac{2\dot{\varepsilon }{{w}^{2}}}{\sqrt{{{\rho }_{{m}}}}a'{{b}^{2}}{{\nu }_{\text{D}}}}$. Employing the dislocation density *ρ*_*m*_ = 10^8^ m^−2^, strain rate $\dot{\varepsilon }$ = 10^−16^ typical for lower mantle conditions and the kink-pair width *w* = 10*b* in agreement with the large-kink approximation (Möller 1978; Caillard and Martin 2003) provides the athermal temperature *T*_*a*_ = 1900 K. Varying *ρ*_*m*_ from 10^7^ to 10^9^ m^−2^; $\dot{\varepsilon }$ from 10 to 14 to 10^−16^ and *w* from 5*b* to 10*b* results in the temperature range *T*_*a*_ of 1800–2050 K. This temperature range appears to be below the temperatures of 3700–4400 K expected in the *D*″ layer (Boehler 2000; Alfè et al. 2002). This implies that [001](010) dislocation glide in MgSiO_3_ post-perovskite would rather occur in the athermal regime in contrast to the bridgmanite phase which is shown to experience high lattice friction opposed to dislocation glide even under lower mantle conditions (Kraych et al. 2016b). Since we have already showed (Goryaeva et al. 2016) that [100] glide is easy in (010), this result strongly highlights the preferential role played by the structural layering along (010) on the rheology of post-perovskite.

## Conclusions

Employing atomic-scale modeling, we characterize the complex core structures of [001] screw and edge dislocations in MgSiO_3_ post-perovskite. Both dissociate in (010) plane into two symmetric partials separated by a stacking fault. Detailed analysis of the dislocation cores reveals their mixed character and the presence of a <100> component. In case of edge dislocations, dissociation occurs into two ½<101> partials, while the <100> component for the screw dislocation core is only 15%. Lattice friction opposed to the [001](010) screw dislocation glide is found to be 3 GPa, while that of edge dislocations is 33% lower. Consequently, plastic deformation is expected to be governed by glide of the screw dislocations.

Relying on the derived characteristics of straight [001](010) screw dislocations, the efficiency of their glide under lower mantle conditions (high *P*–*T* and low strain rates) is further investigated based on the elastic interaction model (Koizumi et al. 1993). We estimate the so-called athermal temperature *T*_*a*_ in conjunction with Orowan’s equation while assuming that kink-pair formation enthalpy 2*H*_2*k*_ scales linearly with *kT* at a given strain rate and dislocation density. At the lower mantle conditions, the athermal temperature *T*_*a*_ is expected to be within 1,800–2,050 K range which infers that [001](010) dislocation glide in MgSiO_3_ post-perovskite at the CMB would occur in the athermal regime.

Full atomic modeling of [100] (Goryaeva et al. 2015b) and [001] dislocation glide (this study) in MgSiO_3_ post-perovskite under lower mantle conditions demonstrates easy slip in (010) plane parallel to the structural layering. Being characterized by significantly different crystal chemistry, Mg–O and Si–O layers in the high-pressure MgSiO_3_ phase lead to a very strong plastic anisotropy with (010) concentrating most of plastic shear. These results agree well with the recent studies which show that observations of seismic anisotropy in regions of the lowermost mantle located beneath subduction zones (Nowacki et al. 2013) and at the edges of the African Large Low Shear Velocity Province (Ford et al. 2015; Ford and Long 2015) are consistent with plasticity models which involve dominant slip on (010) in the post-perovskite.

Our calculations of easy slip on (010) in post-perovskite contrast with recent modeling of dislocation glide in MgSiO_3_ bridgmanite which shows that pressure strongly inhibits dislocation glide in this structure. The bridgmanite to post-perovskite phase transition has thus a strong effect on the rheological properties of the *D*″ layer which has a lower viscosity than the overlying mantle. This has important implications on the dynamics of the mantle since the presence of a low-viscosity post-perovskite enhances heat transfer across the CMB (Nakagawa and Tackley 2011; Benešová and Cižkovà 2016). Further modeling of creep describing the interplay between dislocation glide and point defects diffusion is needed to quantify the viscosity of post-perovskite.
